# Maladie d’Erdheim Chester systémique agressive traitée par la cladribine avec une évolution favorable: à propos d’un cas

**DOI:** 10.11604/pamj.2022.42.32.35085

**Published:** 2022-05-12

**Authors:** Najat Lasri, Fatimaezzahra Lahlimi, Mohammed Ilias Tazi

**Affiliations:** 1 Service d’Hématologie Clinique et de Greffe de Moelle, Centre Hospitalier Universitaire Mohammed VI, Faculté de Médecine et de Pharmacie, Université Cadi Ayyad, Marrakech, Maroc

**Keywords:** Histiocytose non langerhansienne, maladie de Erdheim Chester, atteinte systémique, cladribine, cas clinique, Non-langerhanian histiocytosis, Erdheim-Chester disease, systemic disease, cladribine, case report

## Abstract

La maladie de Erdheim Chester (MEC) est une forme d'histiocytose non langerhansienne à prédominance masculine dont la physiopathologie est mal élucidée. Le tableau clinique est hétérogène. La présence des images de reins chevelus au scanner est pathognomonique. Le diagnostic est anatomopathologique. Nous rapportons le cas d´un patient âgé de 50 ans, qui s´est présenté pour des troubles d´équilibre et une lenteur de parole dans un contexte d´altération d´état général. L´examen clinique a trouvé une ataxie cérébelleuse. Les investigations radiologiques et anatomopathologique ont été en faveur de MEC systémique agressive. Un traitement à base de cladribine a été initié avec une évolution satisfaisante. La MEC est une entité extrêmement rare. Les formes systémiques sont en général de mauvais pronostic et réfractaires au traitement, contrairement à notre cas ayant bien évolué sous cladribine.

## Abstract (en)

Erdheim-Chester disease (ECD) is a form of non-langerhans histiocytosis mainly affecting men, whose pathophysiology is poorly understood. Clinical picture is heterogeneous. The presence of hair kidney sign on Ct scan is pathognomonic. Diagnosis is based on anatomopathological examination. We here report the case of a 50-year old patient presenting with disorders of equilibrium and slow speech as well as general health deterioration Clinical examination showed cerebellar ataxia. Radiological and pathological investigations showed aggressive systemic ECD. cladribine-based treatment was initiated with satisfactory outcome. ECD is an extremely rare disease. Systemic forms are generally associated with poor prognosis and are refractory to treatment, unlike in the case of our patient who was treated with cladribine with good outcome.

## Introduction

La maladie de Erdheim Chester est une histiocytose non langerhansienne très rare à prédominance masculine [1]. La physiopathologie de cette affection est mal élucidée [1,2]. Le pronostic dépend des organes atteints. Les traitements de choix incluent la cladribine [3]. Peu de données concernant le profil thérapeutique et évolutif existent dans la littérature vu l´extrême rareté des séries. Nous rapportons le cas d´un patient âgé de 50 ans diagnostiqué et traité pour maladie de Erdheim Chester au service d´hématologie clinique et de greffe de moelle du Centre hospitalier universitaire Mohammed VI de Marrakech.

## Patient et observation

**Informations du patient:** patient âgé de 50 ans ayant comme antécédent un diabète type II sous insulinothérapie. Il s´est présenté pour des troubles d´équilibre (troubles de marche, chutes fréquentes, maladresse dans les mouvements, vertiges) et lenteur de parole sans autres symptômes associés, remontant à 15 mois avant l´admission.

**Résultats cliniques:** l´examen clinique a trouvé un performance status à 3, un syndrome cérébelleux statique et cinétique fait d´ataxie cérébelleuse, hypermétrie, et dysarthrie, sans autres anomalies associées.

**Figure 1:**
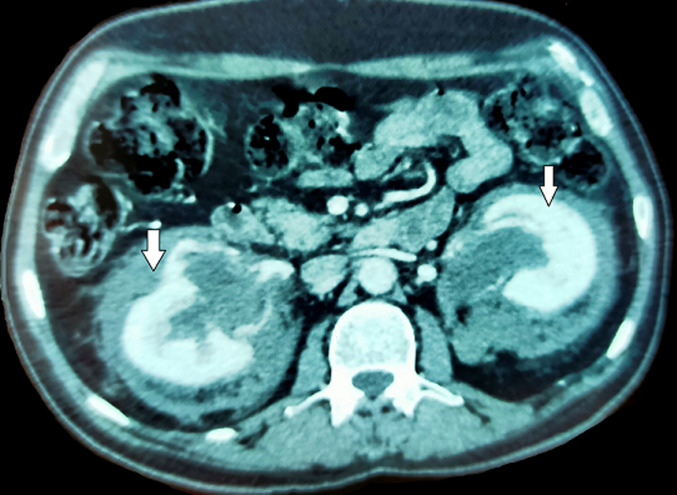
aspect des reins chevelus sur fibrose rétropéritonéale engainante étendu aux sinus rénaux chez notre patient (flèche blanche), cet aspect scanographique est caractéristique de la maladie de Erdeim Chester

**Démarche diagnostique:** l´IRM cérébrale a objectivé une granulomatose inflammatoire (hypersignal T 2, isosignal T 1) au niveau du vermis, hémisphères cérébelleux et de la région hippocampique. Le scanner cervico-thoraco-abdomino-pelvien a montré un aspect de reins chevelus sur fibrose rétropéritonéale engainante étendu aux sinus rénaux (Figure 1), avec dilatation des cavités pyélocalicielles et urétéro-hydronéphrose bilatérale, un épaississement mural péri-aortique, atteinte osseuse à type d´ostéosclérose et condensation en verre dépoli du massif facial, sternum, métaphyses des humérus et des fémurs, ainsi que des micronodules sous pleuraux. La biopsie fémorale gauche a été en faveur de la maladie de Erdheim Chester, avec des logettes osseuses siège d´un infiltrat inflammatoire fait de lymphocytes et de rares polynucléaires neutrophiles, mêlé à de nombreuses cellules vacuolaires d´allure histiocytaires, avec présence de turgescence endothéliale et de suffisions hémorragiques, l´immunohistochimie a objectivé une expression cytoplasmique histiocytaires de CD 68. La mutation BRAF est non réalisable dans notre contexte. La numération sanguine a été normale. La ponction lombaire exploratrice n´a pas montré d´anomalies. Un syndrome inflammatoire biologique a été noté avec une CRP à 86mg/l. le bilan endocrinien (lipidique, thyroïdien, glycémique) ainsi que la fonction rénale et hépatique n´ont pas montré d´anomalies. Le diagnostic de maladie de Erdheim Chester systémique symptomatique dans sa forme agressive avec atteinte neurologique, rétropéritonéale, rénale et osseuse a été retenu.

**Intervention thérapeutique:** le patient a été inclus dans un protocole à base de cladribine à raison de 14mg/kg/jour de J1 à J5 tous les 28 jours. Il a reçu 6 cycles thérapeutiques au total, en parallèle avec un traitement symptomatique (kinésithérapie avec rééducation à la marche et orthophonie).

**Suivi et résultats:** une bonne amélioration clinique au décours du traitement a été notée avec une régression du syndrome cérébelleux et disparition de la dysarthrie. Le scanner cérébral et thoraco-abdomino-pelvien d´évaluation en fin du traitement ont montré une disparition complète des lésions neurologiques, une régression d´infiltration périrénale avec une persistance d´ostéocondensation osseuse minime uniquement au niveau maxillaire et des têtes humérales. Le patient est actuellement sous surveillance avec un recul de 7 mois.

## Discussion

La maladie de Erdheim Chester (MEC) est une forme d'histiocytose non langerhansienne, multi-systémique dont la physiopathologie est mal élucidée. Elle fut découverte en 1930 par Jakob Erdheim et Williams Chester. Les atteintes les plus fréquentes sont osseuse, rénale, oculaire, endocrinienne, neurologiques, et cardiovasculaires [1]. L´atteinte est masculine dans 70 % des cas. Elle est décrite dans tous les types d´âge avec un pic entre 50 et 70 ans. Le délai de consultation dépasse souvent une année parallèlement à notre cas [2,3]. Les signes cliniques dépendent des organes atteints (Tableau 1) [1]. Les formes indolentes existent avec de simples lésions osseuses asymptomatiques. Les formes systémiques sont agressives, avec un risque d´engagement du pronostic vital en cas d´atteinte des organes nobles. Les manifestations osseuses sont fréquentes et pathognomoniques dans la MEC, elles sont rapportées dans 40 % des cas selon Haroche *et al*. Dans une série de 53 patients, elles sont asymptomatiques dans 50 % des cas des formes disséminées (parallèlement à notre patient) , elles touchent particulièrement les diaphyses et métaphyses des os longs. L´imagerie est caractéristique avec présence d´ostéosclérose et de condensations osseuses au scanner, et une hyperfixation manifeste à la scintigraphie osseuse [1,4]. L´atteinte du système nerveux central, présente chez notre patient est décrite dans 40 à 50 % des cas selon les séries. Les localisations les plus fréquentes sont hypothalamo-hypophysaire, pituitaire, cérébelleuse (constatée chez notre patient) et méningées.

**Tableau 1:** fréquence des atteintes cliniques dans la maladie de Erdheim Chester selon Haroche *et al*. [1] (série de 53 patients)

Atteinte Clinique	Fréquence(%)
Os	40
Aorte	66
Péricarde	42
Rétro-orbitaire	25
Diabète insipide	25
Xanthelasma	28
Fibrose rétropéritonéale	50
Système nerveux central	51
Poumon	43

Le tableau clinique est non spécifique et marqué par un handicap majeur. L´imagerie montre une atteinte multifocale et non spécifique [1,2]. L´atteinte hypophysaire est responsable des endocrinopathies notamment l´hypogonadisme, le diabète insipide et l´hyperprolactinémie. L´infiltration rétro-orbitaire et l´atteinte du massif facial est concomitante dans la majorité des cas avec l´atteinte neurologique et permet d´évoquer le diagnostic [5]. La fibrose rétropéritonéale est décrite dans 1/3 des cas. Elle touche souvent les glandes surrénales, les reins (aspect de reins chevelus) et les voies excrétrices avec risque d´insuffisance rénale obstructive et HTA réno-vasculaire [4,6]. Les atteintes cardiaques peuvent être responsables d´un tableau d´insuffisance cardiaque aigue. Elles sont à type de péricardite exposant au risque de tamponnade, cardiomyopathie hypertrophique et troubles de conduction [2]. Sur le plan pulmonaire les infiltrats interstitiels et les épanchements pleuraux liquidiens sont fréquents. La localisation cutanée se manifeste essentiellement par des xanthélasmas [1,2]. Le biopsie d´organe atteint avec étude anatomopathologique confirme le diagnostic en montrant une prolifération histiocytaire au sein de fibrose faite de xantogranulome avec un aspect vacuolisé des cellules. L´immunohistochimie montre une expression du CD 68. La négativité de CD1a permet d´écarter l´histiocytose langerhansienne. Sur le plan moléculaire, la mutation BRAF est rapportée dans plus de 50% des cas [4]. Le diagnostic différentiel se fait avec l´histiocytose langerhansienne, la maladie de Rosai- Dorfman, maladie de Gaucher, la maladie de Niemann-Pick et la maladie de Fabry [1, 2].

Il n´existe pas de consensus thérapeutique concernant la prise ne charge de MEC dans la littérature vu la rareté des séries. Les formes asymptomatiques sont sujettes à une simple surveillance. Pour les formes symptomatiques et asymptomatiques avec localisation neurologique, le traitement de première ligne repose sur l´interféron Þ. Notre patient est traité en première ligne par la cladribine, malgré que cette modalité thérapeutique soit rapportée parmi les choix de 2^e^ ligne, de plus en plus d´auteurs ont démontré son efficacité en première ligne thérapeutique parallèlement à notre cas [3]. Pour les formes réfractaires ou progressive les choix de 2^e^ ligne thérapeutique incluent l´anakinra (inhibiteur du récepteur d´IL1), le vemurafénib (inhibiteur d BRAF), l´infliximab et l´imatinib. La corticothérapie et la polychimiothérapie peuvent être également utilisée dans les traitements de rattrapage de MEC, les molécules les plus utilisées étant: la prednisone, la vinblastine, la vincristine, l´étoposide, la mercaptopurine, le cyclophosphamide, le méthotréxate, la doxorubicine et [2,7,8]. Le pronostic de MEC dépend du caractère symptomatique ou non de la maladie. L´évolution des formes symptomatiques et en fonction des organes atteints. L´atteinte neurologique est un facteur de mauvais pronostic influençant la survie [5]. Selon les séries, la survie globale à 5 ans dans la MEC peut atteindre les 60%, la mortalité est estimée à 26% dans la série de Haroche *et al*. [1,2]. La particularité de notre cas réside dans l´évolution favorable sous traitement avec un recul satisfaisant malgré le caractère agressif de la maladie.

**Point de vue de la patiente:** le long du traitement, l´évolution a été favorable, le patient a ressenti une bonne amélioration à la fin du traitement.

**Consentement éclairé:** nous avons obtenu le consentement éclairé du patient pour utiliser l´image scanographique dans ce rapport de cas.

## Conclusion

La MEC est une entité très rare. Le pronostic dépend des organes atteints. La cladribine en première ligne thérapeutique peut être comme un choix thérapeutique pour le traitement de cette hémopathie dans notre contexte.
